# Corneal confocal microscopy in the evaluation of immune-related motor neuron disease syndrome

**DOI:** 10.1186/s12883-022-02667-5

**Published:** 2022-04-11

**Authors:** Lin Jiao, Yuanjin Zhang, Haikun Wang, Dongsheng Fan

**Affiliations:** 1 Department of Neurology, Peking University Third Hospital, Beijing, China; 2 Beijing Municipal Key Laboratory of Biomarker and Translational Research in Neurodegenerative Diseases, Beijing, China; 3 Department of Ophthalmology, Peking University Third Hospital, Beijing, China

**Keywords:** Motor neuron disease, Corneal confocal microscopy, Langerhans cell, Inferior whorl length, Immunity

## Abstract

**Background:**

To investigate the sensitivity and specificity of corneal confocal microscopy (CCM) in the diagnosis of immune-related motor neuron disease syndrome and evaluation of the response to immunosuppressive therapy.

**Methods:**

Seventy-two patients with clinical manifestations of motor neuron disease (MND) were analysed. According to whether they had concomitant rheumatic immune disease or rheumatic immune antibody abnormalities, they were divided into an MND group (33 patients) and an immune-related MND syndrome group (39 patients). Another 10 healthy adults were selected as the control group. All individuals were examined by CCM.

**Results:**

For Langerhans cell(LC) density, the area under the receiver operating characteristic(ROC)curve was 0.8, the best cut-off was 67.7 cells/mm2, the sensitivity was 79.5%, and the specificity was 72.7%. For inferior whorl length (IWL), the area under the ROC curve was 0.674, the best cut-off was 17.41 mm/mm^2^, the sensitivity was 69.2%, and the specificity was 66.7%. After immunosuppressive therapy in 5 patients with immune-related MND syndrome, the LCD was significantly reduced (*P* < 0.05), and there was no statistically significant change in the IWL (*P* > 0.05).

**Conclusion:**

The LC density and IWL are ideal for distinguishing MND from immune-related MND syndrome. The LC density reflects the immunotherapy response sensitively.

## Introduction

Amyotrophic lateral sclerosis (ALS) is a progressive neurodegenerative disorder primarily involving motor neurons in the cerebral cortex, brainstem and spinal cord. ALS is the most common form of motor neuron disease (MND) [1]_._ Immune-related MND syndrome has gradually attracted attention in recent years, but the diagnosis is difficult, which leads to immature or delayed immunotherapy. It is particularly important to explore sensitive examination techniques [2]. Corneal confocal microscopy (CCM) is a noninvasive imaging method that can be used to study the cornea at the cellular level, mainly for the ophthalmic branch of the trigeminal nerve, including corneal nerve fibres and corneal epithelial Langerhans cells [3, 4]. By using CCM to observe Langerhans cells, we can visualize the degree of neuroinflammatory immune response activation. This study intends to explore the diagnostic value of CCM in immune-related MND syndrome and its utility in determining immunosuppressive therapy response.

## Subjects and methods

### Subject selection

A total of 72 patients who were clinically diagnosed with MND were recruited at Peking University Third Hospital. From December 2019 to October 2020, 33 ALS patients (24 male and 9 female; mean age 48.6 ± 11.1 years), 39 immune-related MND syndrome patients (18 men and 21 women; mean age 55.0 ± 8.8 years), and 10 healthy controls (4 men and 6 women; mean age 43.8 ± 20.5 years) were recruited from individuals who visited the physical examination centre for health check-ups. Five patients with immune-related MND syndrome were treated with immunosuppressive therapy and then assessed and followed up with CCM. Four patients were treated with IVIG and were followed up with for 2 weeks to 1 month. One of these patients also received hydroxychloroquine. Another patient was treated with hydroxychloroquine and eilamod and followed up with for 6 months. Both eyes of each subject were examined by CCM.

### Inclusion and exclusion criteria

Inclusion criteria: the diagnosis of ALS was based on the EI Escorial diagnostic criteria revised in 1994 [5]. At present, there is no unified definition or diagnostic standard for immune-related MND syndrome. In our research, patients were considered to have immune-related MND syndrome if they met the clinical, electrophysiological and neuroimaging standards of ALS but had abnormal rheumatic immune antibodies or concomitant rheumatic immune diseases [6],which may be related to the development of an ALS phenotype. We do not emphasise the requirement for rheumatic immune symptomsis. In our study, 9/39 patients had symptoms of dry mouth.Rheumatic immune indicators were as follows:anti mitochondrial antibody(1/39),anti centromere antibody (2/39),anti histone antibody (1/39),anti nuclear antibody (17/39),anti SSA (6/39),anti SSA52 (8/39),anti PM SCL (3/39),anti ScL-70 (2/39),rheumatoid factor (2/39),anti cardiolipin antibody (4/39),anti RNP (2/39),anti Jo-1 (2/39),anti AMA (2/39),anti PCNA (1/39),and anti dsDNA (1/39).The diagnosis of rheumatic immune diseases was made by relevant experts in rheumatology. Normal adults from the health examination centre were selected as the control group. The exclusion criteria included the following: had ocular diseases known to affect corneal nerve and dendritic cell status (e.g., dry eye disease,acute eye infection,ocular trauma), abnormal glycosylated haemoglobin (HbA1c > 6%), received ocular laser treatment and surgery within 6 months prior to the screening, and had a history of contact lens use in the past three months.

### Specimen collection and detection

At the screening (baseline) and the end of the treatment, both eyes of each enrolled patient were examined by the same qualified, treatment-blinded optometrist using laser corneal confocal microscope (Heidelberg Retinal Tomograph III Rostock Cornea Module, Heidelberg, Germany) to capture CCM images of the inferior whorl (IW) area of their cornea. The subject's eyes were anaesthetized using a drop of 0.4% benoxinate hydrochloride, and Viscotears was used on the front of the eye for lubrication [4]. Three images from the IW area at the level of the subbasal nerve plexus were selected based on their quality and variability. The two-dimensional image captured by CCM had a resolution of 384 × 384 pixels in a 400 × 400 mm2 area, a lateral spatial resolution of 0.5 mm, and a depth of resolution of 1–2 mm. Scanning of the subcorneal basal nerve plexus around the central cornea identified a unique vortex area [7] (between 2.18 and 2.92 mm from the corneal vertex; some of the distal parts of the basal fibres in the cornea had fused together to form a spiral clockwise or counterclockwise pattern). Generally, the cornea was examined by "Z" scanning.

### Observation indicators

ImageJ 1.8.0 was used for analysis, and the plug-in Neuron J was used to track, quantify and analyse the following parameters: Langerhans cell density (LC density; the total number of Langerhans cells per square millimetre in the inferior whorl region) and inferior whorl length (IWL; the total length of nerves per square millimetre in the inferior whorl region) [8, 9]. In CCM images, LCs present as bright corpuscular particles with undefined dendritic cell morphology and a diameter of up to 15 μm,specifically, presented as individual cell bodies without processes, cells bearing dendrites and cells arranged in a network via long interdigitating dendrites [10]. LCs presented as either large cells bearing long processes or smaller cells lacking cell dendrites, most supposedly indicating mature and immature phenotypes, respectively [11].The cells without dendrites most likely represent the immature phenotype of dendritic cells and might still be motile, whereas the cells bearing dendrites are mature and mostly stationary in nature [12].

We chose the IW as our observation indicator because it has been reported that IWL reduction occurs earlier than central corneal nerve fibre reduction and corneal nerve fibre damage is more prominent with IW [8]. In addition,because of its unique pattern, it has been suggested that the IW may be a more reliable landmark for longitudinal and interventional assessment of the corneal subbasal nerve plexus [13].The IW has been identified previously as an area with a vortex-like pattern located inferior and slightly nasal to the corneal apex [14].Its characteristic appearance makes it an ideal anatomical landmark for consistent scanning in the cornea.

### Statistical processing

Analysis was carried out using SPSS 26.0. The quantitative data were tested for homogeneity of normality. Normally distributed variables were expressed as the means ± standard deviation and were compared using Student’s t test. Nonnormally distributed variables were expressed as the median (interquartile range) and were compared using Mann–Whitney U tests. The qualitative data were expressed as the number of participants, and the chi-square test was used for comparisons between groups. ROC curves were drawn, and the sensitivity and specificity of diagnostic indicators were calculated. The CCM parameters (LC density and IWL) of the same patient before and after immunosuppressive treatment were compared by paired t test.All tests were two-tailed (α = 0.05), and a P value < 0.05 indicated statistical significance.

## Results

### Comparison of CCM parameters of the control group, ALS group and immune-related MND syndrome group

**Fig. 1:**
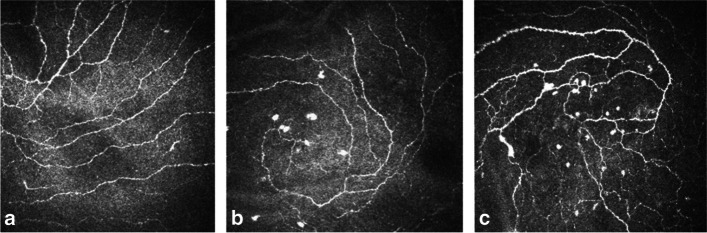
CCM images of the inferior whorl. Subbasal nerve plexus in a healthy control participant (**a**), a patient with ALS (**b**) and a patient with immune-related motor neuron disease syndrome (**c**)

**Fig. 2:**
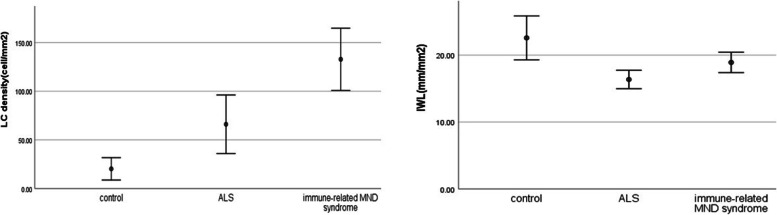
Group comparison of CCM parameters

The LC density values of the ALS group (45.8(54.1)) and the immune-related MND syndrome group (104.1(81.2)) were significantly higher than that of the control group (21.8(17.1)) (*P* < 0.05). The LC density of the immune-related MND syndrome group was higher than that of ALS patients (*P* < 0.05); the IWL values of the ALS group (16.4 ± 3.9) and immune-related MND syndrome group (18.9 ± 4.7) were lower than that of the control group (22.6 ± 4.6) (*P* < 0.05). The IWL of the immune-related MND syndrome group was higher than that of ALS patients (*P* < 0.05) (Table 1, Fig. 1, Fig. 2).

**Table 1:** Comparison of demographics and CCM parameters

	**Control (*n* = 10)**	**ALS (*n* = 33)**	**Immune-related MND syndrome (*n* = 39)**	**P**
Age	43.8 ± 20.5	48.6 ± 11.1	55 ± 8.8	0.019^*a^
Sex (male:female)	4:6	24:9	18:21	0.076^b^
Diagnostic delay (months)	-	26.29	27	0.321
Site of onset (bulbar/limb)	-	3:30	7:32	0.282
ALSFRS-R	-	39.7 ± 5.4	40.2 ± 6.3	0.321
KCSS	-	2 ± 0.9	1.9 ± 0.5	0.229
ΔFS	-	0.7 ± 0.7	0.4 ± 0.4	0.205
LC density (cells/mm^2^)^**^	21.8(17.1)	45.8(54.1)	104.1(81.2)	< 0.01^*c^
IWL (mm/mm^2^)	22.6 ± 4.6	16.4 ± 3.9	18.9 ± 4.7	0.001^*d^

Data are expressed as means ± standard deviations unless otherwise indicated

*ALSFRS-R* amyotrophic lateral sclerosis functional rating score-revised, *KCSS* clinical severity scale, Δ*FS* disease progression rate

^*^ P < 0.05

^**^ LC density expressed as the median(interquartile)

^a^ ALS and immune-related MND syndrome vs. control; *P* = 0.019; ALS vs. immune-related MND syndrome; *P* = 0.013; control vs. ALS; *P* = 0.127

^b^ ALS and immune-related MND syndrome vs. control; *P* = 0.076

^c^ ALS and immune-related MND syndrome vs. control; *P* = 0.010; ALS vs. immune-related MND syndrome; P < 0.01; ALS vs control; *P* = 0.007

^d^ ALS and immune-related MND syndrome vs. control; *P* = 0.001; ALS vs. immune-related MND syndrome; *P* = 0.011; ALS vs control; *P* = 0.001

### ROC curve analysis of the utility of the LC density and IWL for identifying immune-related MND syndrome

**Fig. 3:**
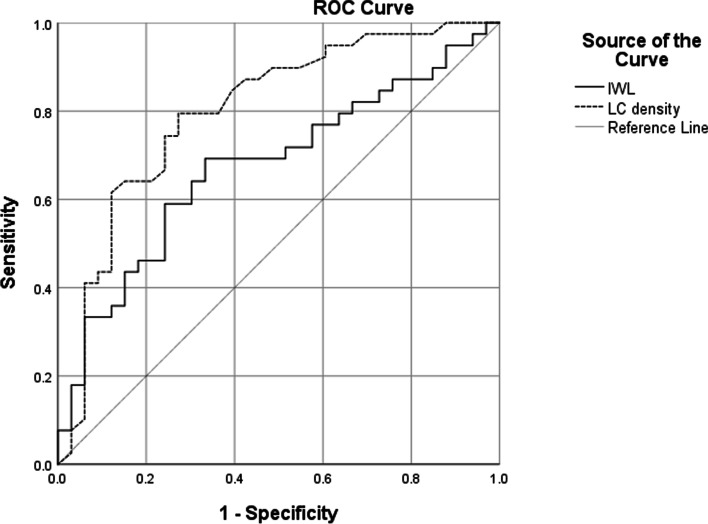
The ROC curves for the LC density and IWL for identifying patients with immune-related MND syndrome

The CCM parameters LC density and IWL were used as diagnostic indicators to perform ROC curve analysis. When the LC density was used to identify patients with immune-related MND syndrome, the area under the curve was 0.8 (95% CI 0.694 ~ 0.906).The best cut-off value was 67.7 cells/mm^2^ (sensitivity 79.5%, specificity 72.7%). When IWL was used to identify patients with immune-related MND syndrome, the area under the curve was 0.674 (95% CI 0.55 ~ 0.799). The best cut-off value was 17.4 mm/mm^2^ (sensitivity 69.2%, specificity 66.7%). The LC density had higher sensitivity and specificity than the IWL (Fig. 3).

### Changes in CCM parameters in patients with immune-related MND syndrome after treatment

**Fig. 4:**
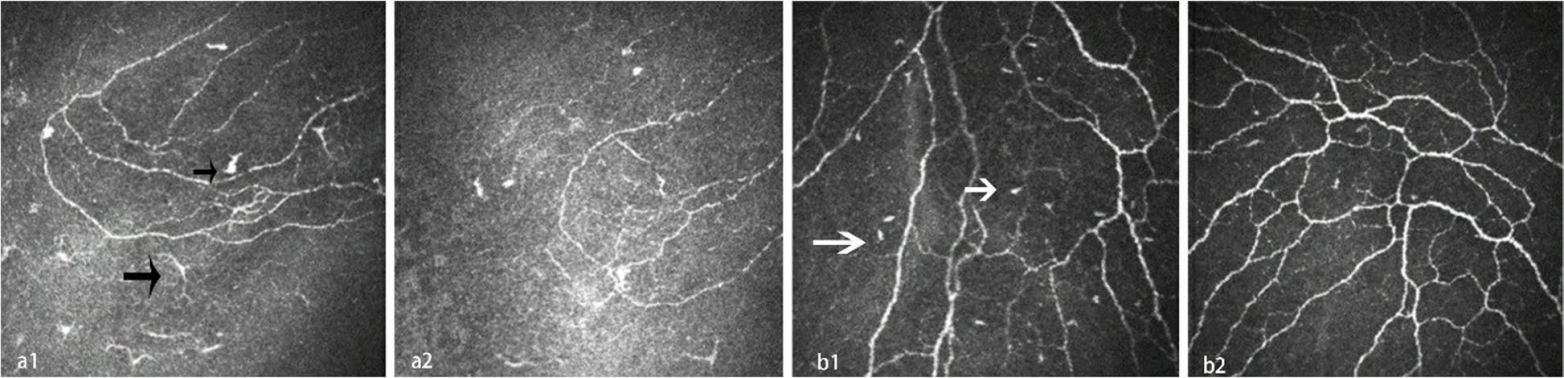
Comparison of inferior whorl area before and after treatment.The Langerhans cells of the inferior whorl were significantly reduced in patients with immune-related motor neuron disease syndrome after receipt of immunosuppressants. a1 and b1 show the CCM images before treatment, and a2 and b2 show the CCM images after treatment of the corresponding patient. Black arrows show mature Langerhans cells, and white arrows show immature Langerhans cells

In the immune-related MND syndrome group, 5 patients were treated with immunosuppressive agents. The patients were followed up for 2 weeks to 6 months, and it was found that the LC density was significantly reduced compared with that before treatment (*P* < 0.05). No significant changes in the IWL were found (*P* > 0.05) (Table 2, Fig. 4). Among these patients,antibodies were re-evaluated after treatment in 3, and there was no change.

**Table 2:** Comparison of CCM parameters before and after treatment with immunosuppressive agents

	**Sex**	**Age**	**Immunosuppressive agents**	**LC density (cells/mm2) (before)**	**LC density(cells/mm2) (after)**	**IWL(mm/mm2) (before)**	**IWL(mm/mm2) (after)**
1	F	51	Hydroxychloroquine, isilamod	87.5	25	15.6	15.3
2	F	54	IVIG	104.2	22.9	20.3	23.8
3	F	57	Hydroxychloroquine, IVIG	272.9	222.9	27.9	26.3
4	F	55	IVIG	241.7	147.9	16.4	15.9
5	F	37	IVIG	39.6	12.5	17.8	16.6
P				0.006^*^		0.968	

^*^P < 0.05

## Discussion

CCM is becoming increasingly important in the diagnosis and management of systemic diseases (such as diabetic peripheral neuropathy and autoimmune diseases) and ophthalmic diseases (such as corneal infection and corneal dystrophy) and is a new imaging technology that can be used to noninvasively observe corneal inflammation and sensory fibres in living tissues [15]. To the best of our knowledge, there are few studies on the application of corneal confocal microscopy in MND. This study focused on immune inflammatory cells and nerve fibres in the inferior whorl area of the cornea. Langerhans cells are the main antigen-presenting cells of the cornea. Morphologically, long, large cells and small cells lacking cell dendrites indicate mature and immature phenotypes, respectively [16]. Cells with dendritic structures were considered mature LCs and those without dendritic structures were considered immature LCs, as per a previously described method. Immature Langerhans cells can capture antigens, while mature Langerhans cells can sensitize naive T cells through the secretion of MHC molecules, interleukin 12, and costimulatory molecules, which are an important part of the immune system [13]. Studies have shown that changes in corneal Langerhans cells are not only related to local inflammation of the eye but also affected by systemic inflammation [17]. The corneal nerve fibres observed by CCM are mainly small sensory fibres of the trigeminal nerve. Studies have shown that the CCM nerve fibre length parameter is a reliable indicator for evaluating corneal nerve fibre damage and repair [18]. Moreover, due to the highly complex pattern of nerves in the inferior whorl area, the main nerves cannot be distinguished from the branches. Therefore, the length of all nerve fibres in the whorl area can be quantified in the form of the IWL. The corneal nerve plexus not only has clinical significance for corneal diseases but also assists in the early assessment of the immune system, early detection of nervous system diseases and detection of late complications of certain systemic diseases, such as diabetes [19, 20].

MND is a serious and fatal disease, and all treatable causes should be sought. At present, there are few studies on rheumatic immune disease combined with MND, but such cases are not uncommon in clinical practice. Some patients can improve after standard immunosuppressive therapy in a short time, and their prognosis is better [21]. This study used ROC curves to evaluate the diagnostic value of the LC density and IWL in the inferior whorl area for identifying immune-related MND syndrome. ROC curve analysis is a method that determines sensitivity and specificity to evaluate the accuracy of diagnostic tests. Studies have revealed that the induction of a certain degree of inflammation not only promotes the regeneration of injured optic nerve axons but also supports the survival of retinal ganglion cells [22]. The results of the present study show that an LC density > 67.7 cells/mm^2^ generated a sensitivity of 79.5% and a specificity of 72.7%, and an IWL > 17.4 mm/mm^2^ generated a sensitivity of 69.2% and a specificity of 66.7%. The LC density had an obviously better diagnostic value than the IWL.

Our research seems to indicate that, compared with traditional rheumatic immune antibodies, Langerhans cells are more sensitive to immune treatment. The patients receiving immunotherapy were followed up for 2 weeks to 6 months, and the LC density was significantly reduced. Furthermore, we assessed the inferior whorl area (which has a unique pattern); it has been shown to be a reliable marker for longitudinal evaluation of the subcorneal basal nerve plexus [23]. At the same time, we rechecked the serum antibodies of 3 patients after immunotherapy, and we did not observe changes in antibodies.Rheumatic immune antibodies often remain unchanged in the short term. A study of 65 patients with systemic lupus erythematosus (SLE) showed that after standard treatment and 10 years of follow-up, the ANA positive rate only decreased from 95.6% to 78.6% [24]. Even if rheumatic immune antibodies can change, long-term, standardized immunosuppressive treatment is often needed. We did not find obvious changes in nerve fibres, indicating that the progression of neurodegeneration may have been under control.

ALS also causes small fibre nerve damage. Bella et al. performed skin biopsies on 51 ALS patients and quantified the density of intraepidermal nerve fibres (IENFs) [25]. The results showed that all patients had a reduced density of IEFNs, indicating that the neurodegenerative process of ALS affects small fibre nerves. Ferrari et al. conducted CCM examination of 8 ALS patients and found that the length of corneal nerve fibres was reduced compared with that in the control group, and it was related to the degree of bulbar involvement [26]. We found that the IWL of patients with MND and immune-related MND syndrome was lower than that of the control group (*P* < 0.05), and it also suggested sensory small fibre nerve changes, which is consistent with the abovementioned literature reports. Furthermore, the IWL of immune-related MND syndrome was higher than that of ALS (*P* < 0.05).We suppose that a certain degree of inflammatory factors may have a protective effect on nerves.A certain density of inflammatory cells can promote corneal nerve regeneration, while excessive inflammation may lead to loss of corneal innervation and subsequent neurotrophic keratopathy [27].

Immune mechanisms may be involved in the pathogenesis of ALS. In animal models, the specific deletion of the C9orf72 gene in mouse myeloid cells leads to lysosome accumulation, a hyperimmune response, and increased expression of the interleukins IL-6 and IL-1β, which changes the immune function of these cells and then causes neurodegeneration [28]. Lu CH et al. found that in ALS patients, the levels of TNF-α, IL-1β, IL-2, IL-8, IL-12, IL-4, IL-5, and IL-10 were significantly higher than those of the control group, suggesting that most inflammatory factors of the T-cell immune response may be involved in the pathogenesis of ALS [29]. The results of this study found that the LC density of ALS patients was higher than that of the control group (*P* < 0.05), which provides additional evidence for the immune mechanism of ALS.

However, it must be noted that the number of research groups was not large enough. In addition, CCM can only assess the correlation between corneal nerve plexus pathology and disease, and the pathogenesis of the disease needs to be further studied.

In conclusion, this study demonstrated that CCM parameters, especially the LC density, are potential diagnostic tools for immune-related MND syndrome. The LC density is more sensitive to immunosuppressive agents. In addition, our study also provides some evidence for the immune mechanism and small fibre nerve damage of ALS.

## Data Availability

The datasets generated and/or analysed during the current study are not publicly available due to privacy or ethical restrictions. But are available from the corresponding author on reasonable request.

## Abbreviations

CCM: Corneal confocal microscopy
MND: Motor neuron disease
LC: Langerhans cell
ALS: Amyotrophic lateral sclerosis
IWL: Inferior whorl length
ALSFRS-R: Amyotrophic lateral sclerosis functional rating score-revised
ROC: Receiver operating characteristic
